# Research progress on the role of bypass activation mechanisms in resistance to tyrosine kinase inhibitors in non-small cell lung cancer

**DOI:** 10.3389/fonc.2024.1447678

**Published:** 2024-11-08

**Authors:** Ziyang Jiang, Zhihan Gu, Xiaomin Yu, Tao Cheng, Bofu Liu

**Affiliations:** ^1^ Department of Emergency Medicine and Laboratory of Emergency Medicine, West China Hospital, Sichuan University, Chengdu, China; ^2^ Department of Emergency Medicine, West China Hospital, Sichuan University, West China School of Nursing, Sichuan University, Chengdu, China; ^3^ Institute of Disaster Medicine, Sichuan University, Chengdu, China; ^4^ Nursing Key Laboratory of Sichuan Province, West China Hospital, Chengdu, China

**Keywords:** tyrosine kinase inhibitors, NSCLC, bypass activation resistance, targeted therapy, gene ontology

## Abstract

The clinical application of small molecule tyrosine kinase inhibitors (TKIs) has significantly improved the quality of life and prognosis of patients with non-small cell lung cancer (NSCLC) carrying driver genes. However, resistance to TKI treatment is inevitable. Bypass signal activation is one of the important reasons for TKI resistance. Although TKI drugs inhibit downstream signaling pathways of driver genes, key signaling pathways within tumor cells can still be persistently activated through bypass routes such as MET gene amplification, EGFR gene amplification, and AXL activation. This continuous activation maintains tumor cell growth and proliferation, leading to TKI resistance. The fundamental strategy to treat TKI resistance mediated by bypass activation involves simultaneously inhibiting the activated bypass signals and the original driver gene signaling pathways. Some clinical trials based on this combined treatment approach have yielded promising preliminary results, offering more treatment options for NSCLC patients with TKI resistance. Additionally, early identification of resistance mechanisms through liquid biopsy, personalized targeted therapy against these mechanisms, and preemptive targeting of drug-tolerant persistent cells may provide NSCLC patients with more sustained and effective treatment.

## 1 Introduction

Lung cancer is one of the leading causes of cancer-related deaths worldwide. According to the 2022 Global Cancer Research Report, nearly 20 million people were newly diagnosed with cancer globally in 2022, with approximately 8 million deaths. Primary lung cancer is the most common among all newly diagnosed cancer cases, with about 2.5 million new cases, accounting for 12.4% of global cancer cases (1). In the United States, thanks to advancements in cancer screening, diagnosis, and treatment technologies, the cancer mortality rate decreased annually by approximately 1.2% from 2016 to 2020, with a particularly noticeable decline in lung cancer mortality (2). The quality of life and prognostic outcomes of lung cancer patients have been greatly improved by the use of TKIs drugs.

Based on histological types, lung cancer can be classified into non-small cell lung cancer (NSCLC) and small cell lung cancer (SCLC), with NSCLC accounting for 80%-85% of all lung cancers (3–5). Current research indicates that more than two-thirds of NSCLC cases carry tumor driver genes (6, 7), including epidermal growth factor receptor (EGFR) gene (10%-15% in Western populations and 30%-50% in East Asian populations) (8, 9), Kirsten rat sarcoma viral oncogene (KRAS) (15%-20%) (10), anaplastic lymphoma kinase (EML4-ALK) (3%-7%) (10, 11), and c-ros oncogene 1 (ROS1) (1%-2%) (12) etc. The U.S. Food and Drug Administration (FDA) has approved various tyrosine kinase inhibitors (TKIs) for clinical treatment of NSCLC with these driver genes, such as first-generation EGFR TKIs [Gefitinib (13, 14) and Erlotinib (15)], second-generation EGFR TKIs [Afatinib (16) and Dacomitinib (17)], third-generation EGFR TKIs [Osimertinib (18–20)], first-generation ALK TKIs [Crizotinib (21–23)], second-generation ALK TKIs [Ceritinib (24–26), Alectinib (27), and Brigatinib (28)], and third-generation ALK TKIs [Lorlatinib (29, 30)] (see **Table 1**). The application of TKI drugs has significantly improved the prognosis of NSCLC patients with driver genes. Unfortunately, most patients develop resistance to TKI drugs after 1 to 2 years of treatment, causing tumor progression and making it difficult for patients to achieve long-term benefits from TKI therapy (31–33).

**Table 1:** FDA-approved kinase inhibitors for advanced NSCLC treatment.

Name	Approval Year	Target	Indication
Gefitinib	2003	EGFR	EGFR-mutant NSCLC
Erlotinib	2004	EGFR	EGFR-mutant NSCLC
Crizotinib	2011	ALK and ROS1	ALK and ROS1 fusion-positive NSCLC
Afatinib	2013	EGFR, HER2, and HER4	EGFR-mutant NSCLC
Trametinib	2013	MEK1/2	BRAF-mutant NSCLC
Dabrafenib	2013	BRAF	BRAF-mutant NSCLC
Ceritinib	2014	ALK, IGF-1R, and ROS1	ALK fusion-positive NSCLC
Osimertinib	2015	EGFR	EGFR-mutant NSCLC
Alectinib	2015	ALK, RET	ALK fusion-positive NSCLC
Brigatinib	2017	ALK, ROS1, IGF-1R, FLT3, and EGFR	ALK fusion-positive NSCLC
Dacomitinib	2018	EGFR, HER2, and HER4	EGFR-mutant NSCLC
Lorlatinib	2018	ALK and ROS1	ALK fusion-positive NSCLC
Entrectinib	2019	TRKA/B/C, ROS1, and ALK	NTRK fusion-positive NSCLC
Capmatinib	2020	MET	MET exon 14 skipping mutant NSCLC
Selpercatinib	2020	RET	RET fusion-positive NSCLC
Pralsetinib	2020	RET	RET fusion-positive NSCLC
Tepotinib	2021	MET	MET exon 14 skipping mutant NSCLC
Mobocertinib	2023	EGFR	EGFR-mutant NSCLC
Amivantamab	2023	EGFR	EGFR-mutant NSCLC

The mechanisms of TKI resistance in NSCLC mainly include primary resistance and acquired resistance (31, 34, 35). Studies have shown that 4%-10% of NSCLC patients exhibit primary resistance to TKIs, which may be due to the presence of other non-sensitive mutations in the target gene, although the exact molecular mechanisms are not fully understood (31). Over 90% of TKI resistance is acquired resistance, which includes mechanisms such as secondary mutations in the target gene, bypass activation, and histological transformation (31, 34). In patients with EGFR mutations, approximately 60% of TKI resistance is caused by secondary mutations in the EGFR gene, while about 20% is due to bypass activation (31, 34, 35). For patients with ALK gene fusions, 28%-50% of TKI resistance is due to gene mutations in the ALK kinase domain, and 40%-50% of patients develop resistance to ALK TKIs through bypass activation (31, 34, 35). Secondary mutations in the target gene can impede the binding of TKI drugs to the kinase by altering the conformation of the tyrosine kinase and/or changing the affinity between the kinase and ATP, resulting in the loss of the drug’s cytotoxic effect on tumor cells (36, 37). New generations of TKI drugs are typically designed to target secondary mutation sites that confer resistance to previous generations, allowing patients with secondary mutations to continue benefiting from treatment. For example, in EGFR-mutant NSCLC patients, the EGFR T790M mutation is a significant cause of resistance to first- and second-generation EGFR TKIs (38, 39). Third-generation EGFR TKIs, such as Osimertinib, are designed to target the EGFR T790M mutation, significantly improving the prognosis of NSCLC patients with this mutation (19). In bypass activation-mediated TKI resistance, tumor cells evade the cytotoxic effects of TKI drugs through continuous activation of bypass signaling pathways. The mechanisms of bypass activation-mediated resistance are complex, currently there is no standard clinical treatment for bypass activation resistance. Therefore, this review will summarize the research progress on bypass activation mechanisms in NSCLC TKI resistance, aiming to provide new insights and clues for future research on NSCLC TKI resistance mechanisms and the development of combination treatment strategies that can overcome or reverse resistance.

## 2 Overview of bypass activation resistance

Tumor cells maintain proliferation and differentiation through a coordinated network of intracellular signaling pathways. When the primary signaling pathways in tumor cells are inhibited by TKI drugs, the tumor cells can reactivate key downstream effectors required for cell survival and proliferation through parallel signaling pathways of other receptor tyrosine kinases (RTKs). This allows the tumor to bypass the inhibition of the driver gene by the TKI drug and continue to survive and grow (34, 40). This mechanism is known as bypass activation. In 2007, Engelman et al. (41) first described this resistance mechanism. They found Mesenchymal to Epithelial Transition Factor(MET) gene amplification in about 20% of patients with EGFR TKI resistance. MET gene amplification bypasses the EGFR signaling pathway by mediating HER3 phosphorylation, thereby reactivating the PI3K/AKT signaling pathway (41).

Theoretically, the presence of each driver gene in NSCLC is mutually exclusive, but under TKI drug treatment, driver genes often operate in a complementary manner to sustain cell survival and growth. MET gene amplification, which mediates resistance to EGFR TKIs, is one such example. Additionally, in EML4-ALK fusion NSCLC, approximately 40% of Crizotinib-resistant cases exhibit activation of the EGFR signaling pathway (42–44). In these cases, EGFR pathway activation is often due to EGFR gene amplification rather than EGFR gene mutation.

Besides MET gene amplification and EGFR pathway activation, other bypass activation pathways can mediate NSCLC TKI resistance. These include AXL activation (45), insulin-like growth factor receptor (IGF-1R) activation (46), and human epidermal growth factor receptor 2 (HER2) amplification (47). Common bypass activation pathways in NSCLC TKI resistance are summarized in **Table 2**.

**Table 2:** Common bypass activation pathways in NSCLC TKI resistance.

TKI Type	Bypass Activation Pathway	Proportion
Resistance to First- and Second-Generation EGFR TKIs		
	MET gene amplification	5%-22%
	HER2 gene amplification	10%-15%
	AXL activation	~20%
	PIK3CA mutation	2%-3%
	BRAF mutation	~1%
	KRAS mutation	~1%
Resistance to First-Line Treatment with Third-Generation EGFR TKIs		
	MET gene amplification	7%-15%
	HER2 gene amplification	1%-2%
	PIK3CA mutation	7%
	BRAF mutation	3%
	KRAS mutation	3%-4%
Resistance to Second-Line Treatment with Third-Generation EGFR TKIs		
	MET gene amplification	6%-26%
	HER2 gene amplification	3%-5%
	PIK3CA amplification	4%-11%
	BRAF mutation	3%
	KRAS mutation	2%-8%
Resistance to ALK TKIs		
	EGFR activation	30%-44%
	MET gene amplification	15%
	KIT gene amplification	11%
	PIK3CA mutation	3%
	BRAF mutation	3%
	NRAS mutation	3%
	FGFR2 mutation	3%
Resistance to MET TKIs		
	EGFR amplification	13%
	KRAS mutation or amplification	3%
Resistance to RET TKIs		
	MET gene amplification	15%
	KRAS gene amplification	5%

## 3 Common bypass activation pathways mediating NSCLC TKI resistance

### 3.1 MET bypass activation pathway mediating NSCLC TKI resistance

#### 3.1.1 Overview of the MET gene

The MET proto-oncogene is located on human chromosome 7q21-q31, spanning approximately 125 kb and containing 21 exons and 20 introns (48). The protein encoded by the MET gene, c-MET, is a transmembrane receptor tyrosine kinase composed of an extracellular SEMA (Semaphorin) domain, PSI (Plexin Semaphoring Integrin) domain, and IPT (Immunoglobulin Plexins Transcription) domain, a transmembrane domain, and an intracellular juxtamembrane (JM) domain, tyrosine kinase domain, and a carboxy-terminal region. This heterodimer has self-phosphorylation capability (49). Hepatocyte Growth Factor (HGF) is currently the only known high-affinity ligand for c-MET. When the HGF ligand binds to the SEMA domain of c-MET, the intracellular tyrosine residues of c-MET undergo homodimerization and phosphorylation, thereby activating c-MET. This activation subsequently triggers downstream signaling pathways such as RAS/ERK/MAPK, PI3K/AKT, Wnt/β-catenin, and STAT, promoting cell growth, proliferation, migration, angiogenesis, and epithelial-to-mesenchymal transition (EMT) (50, 51). In NSCLC, the abnormal activation of the c-MET signaling pathway mainly includes MET gene amplification (52), MET exon 14 skipping mutations (53), MET gene rearrangements (54, 55), and c-MET protein overexpression (56, 57).

#### 3.1.2 MET gene amplification and NSCLC TKI resistance

MET gene amplification refers to an increase in the copy number of the MET gene (52). This increase is often achieved through gene breakage-fusion-bridge mechanisms that replicate the gene region or local area (58). MET gene amplification leads to overexpression of the c-MET protein, thereby activating downstream signaling pathways. Studies (59) have shown that MET gene amplification can often coexist with other tumor driver genes in NSCLC, such as EGFR and KRAS mutations and ALK and ROS1 fusions, indicating that MET gene amplification may not act as an intrinsic driver factor within tumors. In NSCLC, MET gene amplification is considered the most common mechanism of TKI resistance mediated by bypass activation pathways (31, 32, 34). The frequency of MET gene amplification in various gene mutation types of NSCLC TKI resistance is shown in **Table 3**.

**Table 3:** Frequency of MET gene amplification in NSCLC TKI resistance.

Study	TKI Drug	Sample Size	Detection Method	MET Amplification Frequency
Engelman et al. (41)	Gefitinib	18	Tissue	22%
Bean et al. (60)	First-generation EGFR TKI	43	Tissue	21%
Turke et al. (61)	First-generation EGFR TKI	27	Tissue	15%
Zhang et al. (45)	First-generation EGFR TKI	31	Tissue	19%
Yano et al. (62)	First-generation EGFR TKI	93	Tissue	9%
Sequist et al. (63)	First-generation EGFR TKI	37	Tissue	5%
Yu et al. (64)	First-generation EGFR TKI	75	Tissue	5%
Suda et al. (65)	Gefitinib	13	Tissue	8%
Arcila et al. (66)	First-generation EGFR TKI	37	Tissue	11%
Cardona et al. (67)	Erlotinib	34	Tissue	9%
Zhang et al. (68)	Abivertinib (second-line)	16	Plasma	6%
Chabon et al. (69)	Rociletinib (second- or third-line)	43	Plasma	26%
Piotrowska et al. (70)	Osimertinib (second- or third-line)	41	Tissue and/or plasma	24%
Mehlman et al. (71)	Osimertinib (second- or third-line)	226	Tissue and/or plasma	11%
Oxnard et al. (72)	Osimertinib (second-line)	41	Tissue and/or plasma	10%
Le et al. (73)	Osimertinib (first- or second-line)	42	Tissue and/or plasma	14%
Papadimitrakopoulou et al. (74)	Osimertinib (second-line)	73	Plasma	19%
Ramalingam et al. (75)	Osimertinib (first-line)	91	Plasma	15%
Dagogo-Jack et al. (76)	ALK TKI	136	Tissue and/or plasma	12% (second-generation); 22% (third-generation)
Lin et al. (77)	RET TKI	18	Tissue and/or plasma	15%

Research has reported that the occurrence rate of MET gene amplification in first-generation EGFR TKI-resistant patients is approximately 5% to 20% (41, 60–67). In the earliest related reports, among 18 NSCLC patients resistant to the first-generation EGFR TKIs erlotinib or gefitinib, MET gene amplification was found in 4 cases (22%) (41). Similarly, in the study by Bean et al. (60), among 43 NSCLC patients who developed resistance to erlotinib or gefitinib treatment, 9 cases (21%) were found to have MET gene amplification, while among 62 NSCLC patients who did not receive any treatment, only 2 cases (3%) carried MET gene amplification. In another study by Yu et al. (64), involved 155 NSCLC patients resistant to gefitinib or erlotinib, only 5% of the patients had MET gene amplification. In the study by Sequist et al. (63), only 5% of the patients resistant to gefitinib or erlotinib showed MET gene amplification. Nevertheless, MET gene amplification has been considered an important marker for bypass activation mediating resistance to first-generation EGFR TKIs. Meanwhile, the c-MET ligand HGF can also mediate resistance of NSCLC to gefitinib through the activation of the PI3K/AKT signaling pathway via GAB1 (Grb2 associated binder 1) (61). In the study by Yano et al. (62), 61% of NSCLC patients exhibited high expression of HGF after acquiring resistance to EGFR TKIs, with high HGF expression occurring in 29% of patients with primary resistance to EGFR TKIs. Additionally, in the study by Zhong et al. (78), approximately 20% of patients with primary resistance to erlotinib or gefitinib had MET gene amplification, indicating that MET gene amplification may also be one of the causes of primary resistance of NSCLC to first-generation EGFR TKIs.

In third-generation EGFR TKI resistance, when osimertinib is used in cases of prior failure of EGFR TKIs in second-line treatment, regardless of whether T790M mutation existed previously in patients, resistance may occur through MET gene amplification-mediated bypass activation pathways. After resistance to osimertinib, the incidence of MET gene amplification can reach 19% (74). When osimertinib is used as first-line treatment, the incidence of MET gene amplification in osimertinib-resistant samples can reach 15% (75). In the study by Oxnard et al. (72), approximately 10% of NSCLC patients with EGFR mutations resistant to osimertinib had MET gene amplification. Similarly, in the study by Le et al. (73), 14% of NSCLC patients developed MET gene amplification after resistance to osimertinib. In this study, MET gene amplification was the most common molecular mechanism leading to osimertinib resistance besides EGFR secondary mutations. In another study, Chabon et al. (69) explored the mechanism of resistance to another third-generation EGFR TKI, rociletinib, in 43 NSCLC patients by detecting circulating tumor DNA (ctDNA), and found that 26% of patients developed resistance to rociletinib through MET gene amplification, which was the most common molecular mechanism of rociletinib resistance in the study. Moreover, in this study, when EGFR T790M and MET gene amplification coexisted, it could mediate primary resistance of NSCLC patients to rociletinib (69). Additionally, MET gene amplification can also mediate primary resistance of EGFR mutated NSCLC to osimertinib (79). It is worth noting that MET gene amplification can coexist with secondary mutations of driver genes in the same resistant sample. Papadimitrakopoulou et al. (74) showed in their study that in osimertinib-resistant cases mediated by MET amplification, approximately 7% of patients simultaneously had EGFR C797S mutation.

Similar to mediating EGFR TKI resistance, MET gene amplification can mediate resistance of NSCLC patients to ALK TKIs by activating the c-MET signaling pathway. Berger et al. (80) reported a case of MET gene amplification occurring in an ALK fusion NSCLC patient after resistance to the first-generation ALK TKI crizotinib. Additionally, Ueda et al. (81) reported a case of MET gene amplification occurring in an ALK fusion NSCLC patient after resistance to the third-generation ALK TKI lorlatinib. In the study by Dagogo-Jack et al. (76), which included 136 NSCLC patients who had received at least one ALK TKI drug and developed resistance, approximately 15% of patients showed MET gene amplification after resistance. Furthermore, studies have reported that overexpression of the c-MET ligand HGF can also mediate resistance of NSCLC cells to ALK TKIs through the activation of the MET bypass signaling pathway (82, 83).

Additionally, in a study, after resistance to RET inhibitors, 15% of RET fusion NSCLC patients developed MET gene amplification, while only 10% of patients developed resistance through secondary mutations in the RET gene, demonstrating that MET gene amplification is also one of the most common causes of resistance to RET inhibitors (77).

Interestingly, whether in EGFR TKI resistance or ALK TKI resistance, the incidence of MET gene amplification in the latest generation TKI resistance is higher than in the previous generation TKI resistance. For example, in NSCLC patients resistant to first-generation EGFR TKIs, the incidence of MET gene amplification is 5%-22% (41, 60–67), while in patients resistant to third-generation EGFR TKIs, the incidence of MET gene amplification can be as high as 10%-26% (69, 72–75); in NSCLC patients resistant to second-generation ALK TKIs, the incidence of MET gene amplification is approximately 12%, whereas in patients resistant to third-generation ALK TKIs, MET gene amplification can reach 22% (76). New generations of TKI drugs often have higher target selectivity and treatment efficacy, which may be an important reason for the change in TKI resistance patterns, but there is currently no relevant research confirming this hypothesis, future studies can further explore this phenomenon.

In summary, MET gene amplification plays an important role in mediating resistance to EGFR and ALK TKIs, especially as the most important bypass activation resistance pathway in the latest generation TKI resistance. In clinical practice, combination therapy using MET inhibitors and EGFR/ALK TKIs may overcome resistance caused by MET gene amplification.

#### 3.1.3 MET gene exon 14 skipping mutation and NSCLC TKI resistance

Under normal circumstances, exon 14 of the MET gene is spliced from both sides of the introns to form mRNA precursor, which mainly encodes the JM domain of MET, containing the Y1003 c-Cbl E3 ubiquitin ligase binding site. Ubiquitination of this site leads to degradation of the c-MET receptor (84). When exon 14 of the MET gene undergoes skipping mutation, the translated c-MET receptor lacks the Y1003 c-Cbl binding site, leading to reduced ubiquitination and slower degradation of the c-MET receptor, thereby sustaining activation of the c-MET signaling pathway (85). The MET gene exon 14 skipping mutation is considered a primary oncogenic driver in NSCLC and does not coexist with other NSCLC driver genes such as EGFR, KRAS, ALK, and ROS1 (86). In a study of 933 non-squamous NSCLC cases, no patients with MET gene exon 14 skipping mutation had mutations in KRAS, EGFR, and ERBB2, or rearrangements in ALK, ROS1, and RET (87). As a primary oncogenic driver, the incidence of MET gene exon 14 skipping mutation in NSCLC is 3%-4% (10, 84, 86, 87).

However, in a recent study, researchers from Memorial Sloan Kettering Cancer Center reported a case of lung adenocarcinoma with EGFR L858R mutation, where MET gene exon 14 skipping mutation (c.2899G>A) occurred after resistance to erlotinib treatment. Subsequent treatment with osimertinib and crizotinib combination therapy led to clinical remission (88). Further research showed that the presence of MET gene exon 14 skipping mutation caused the AKT and ERK downstream signaling pathways of EGFR-mutated lung cancer cells to no longer be inhibited by osimertinib, reducing cell sensitivity to osimertinib by approximately 20% (88). Additionally, Dagogo-Jack et al. (76) also reported a case of lung adenocarcinoma with EML4-ALK fusion after sequential treatment with alectinib and brigatinib, where MET gene exon 14 skipping mutation was detected only in the patient’s plasma cell-free DNA.

#### 3.1.4 MET gene rearrangement and c-MET protein overexpression in NSCLC TKI resistance

MET gene rearrangement refers to fusion rearrangement of the MET gene with other genes, activating the MET signaling pathway. c-MET protein overexpression refers to an increase in c-MET protein expression without MET gene amplification, exon 14 skipping mutation, or rearrangement, resulting in c-MET protein overexpression and activation of the MET signaling pathway. Reports of MET gene rearrangement and c-MET protein overexpression in NSCLC TKI resistance are limited. Dagogo-Jack et al. (76) reported cases of patients resistant to second-generation ALK TKI alectinib and sequential treatment with ceritinib, alectinib, and lorlatinib, all showing ST7-MET gene fusion. *In vitro* experiments confirmed that this fusion gene mediated resistance of H3122 cells to ALK TKIs, and resistance mediated by rearrangement of ST7-MET gene could be reversed by combination therapy with MET inhibitors and ALK TKIs. Xu et al. (56) reported a case of NSCLC with EGFR L858R mutation, where increased c-MET protein expression was detected after resistance to gefitinib treatment without MET gene amplification or mutation, indicating resistance to gefitinib due to c-MET protein overexpression. The patient responded to gefitinib combined with crizotinib therapy. In summary, although the frequency of MET gene rearrangement and c-MET protein overexpression in NSCLC TKI resistance is low, they are also one of the reasons for mediating resistance through bypass activation pathways.

### 3.2 EGFR bypass activation pathways in NSCLC TKI resistance

#### 3.2.1 Overview of the EGFR gene

The EGFR gene is located on the short arm of chromosome 7 (7p12) in humans, spanning approximately 118 kb and comprising 28 exons, with mutations in the tyrosine kinase domain of EGFR primarily occurring in exons 18-21 (89). EGFR is a common receptor tyrosine kinase (RTK) and the first member of the ErbB family. Structurally, EGFR consists of extracellular ligand-binding domains, transmembrane domains, and intracellular tyrosine kinase domains (89). Under normal conditions, EGFR exists as an inactive monomer. Upon binding with ligands such as epidermal growth factor (EGF) or transforming growth factor α (TGFα), the receptor undergoes conformational changes, forming homodimers or heterodimers, thereby activating autophosphorylation of key tyrosine residues in the tyrosine kinase domain. Gene mutations in the EGFR tyrosine kinase domain can also lead to autophosphorylation of tyrosine residues, initiating downstream signaling pathways such as RAS/RAF/MEK/ERK, PI3K/AKT/mTOR, and promoting cell proliferation and growth through cascade reactions (89–91).

#### 3.2.2 EGFR bypass activation in NSCLC TKI resistance

In one study, NSCLC patients with ALK fusion showed EGFR gene activation in 44% of tumor samples after developing resistance to ALK TKI crizotinib (43). It has been reported that EGFR bypass activation pathway-mediated resistance to ALK TKIs remains resistant to newer generations of ALK TKIs, but co-administration of EGFR TKIs effectively enhances the cytotoxic effects of ALK TKIs on resistant cells (92). Additionally, overexpression of EGFR ligands EGF and TGFα has been implicated in the mechanism of EGFR bypass activation-mediated resistance (42, 93). EGFR gene mutations and ALK gene fusion are both important driver genes in NSCLC, typically not co-occurring in the same tumor. However, studies have reported that EGFR gene mutations can appear as a means of activating the EGFR bypass signaling pathway in NSCLC patients resistant to ALK TKIs (44, 94). Similarly, the EGFR bypass activation pathway can mediate resistance of ROS1 fusion NSCLC to ROS1 TKIs (95), which can be reversed by co-administration of ROS1 and EGFR inhibitors (96, 97). Study by Lee C et al. (98) revealed that the mutation in EGFR-KDD conferred resistance to 1st and 2nd generation EGFR TKIs, but is sensitive to 3rd generation EGFR TKIs. Furthermore, research by Guo et al. (99) found that approximately 13% of NSCLC patients resistant to MET inhibitors had EGFR gene amplification, suggesting that EGFR bypass signaling pathway activation may be involved in resistance to MET inhibitors.

In summary, EGFR gene activation serves as an important bypass signaling transduction pathway mediating resistance to ALK, ROS1, and MET TKIs. Combination therapy with EGFR TKIs and ALK/ROS1 TKIs may provide an alternative treatment option for patients with resistance mediated by activation of the EGFR signaling pathway.

### 3.3 AXL bypass activation pathway in TKI resistance

#### 3.3.1 Overview of the AXL gene

The AXL gene is located on chromosome 19 (19q13.2) in humans and consists of 20 exons, encoding a protein of 894 amino acids. The AXL protein encoded by the AXL gene is a member of the Tumor-Associated Macrophage (TAM) RTK family, composed of two extracellular immunoglobulin-like repeat sequences, two type III fibronectin repeat sequences, a transmembrane domain, and an intracellular tyrosine kinase domain (100–102). Activation of the AXL receptor can be achieved through ligand binding, AXL overexpression leading to homodimerization, or heterodimerization with other RTKs, with ligand binding being the primary mode of AXL receptor activation (101–103). Growth arrest-specific protein 6 (GAS6) is considered the sole ligand that binds to the extracellular domain of the AXL receptor (101, 102, 104). Upon binding to GAS6, AXL activates downstream signaling pathways such as PI3K/AKT/mTOR, MAPK/ERK, JAK/STAT, and NF-κB through homodimerization and autophosphorylation, thereby promoting activities including cell proliferation, migration, angiogenesis, and mediating cell resistance (101, 102). It is worth noting that unlike other driver genes such as EGFR, ALK, and MET, the expression regulation of AXL is not mediated by gene mutations, fusions, or amplifications (105) but through mechanisms such as transcription factor activation (106–110), microRNA regulation (111–113), or post-transcriptional translation (114–116).

#### 3.3.2 AXL bypass activation pathway-mediated NSCLC TKI resistance

According to a study, approximately 20% of NSCLC patients who developed resistance to first-generation EGFR TKIs erlotinib or gefitinib showed upregulated AXL expression, and about 25% showed upregulated GAS6 expression (45) In preclinical models, AXL has been shown to mediate resistance of NSCLC cells to first-, second-, and even third-generation EGFR TKIs through activation of bypass signaling pathways such as PI3K/AKT, MAPK/ERK, and NF-κB. Restoring sensitivity of NSCLC cells to EGFR TKIs can be achieved by inhibiting AXL gene expression or using AXL inhibitors (116–120). Studies have also reported that AXL can mediate resistance of NSCLC cells to EGFR TKIs by regulating the expression of microRNAs (121). Epithelial-mesenchymal transition (EMT), a biological process involving the transformation of polarized epithelial cells into mesenchymal cells, is also implicated in EGFR TKI resistance (122–124). Activation of AXL often accompanies the occurrence of tumor cell EMT, promoting resistance of NSCLC cells to EGFR TKIs. Inhibiting AXL in resistant cells can reverse the EMT process, overcoming resistance (125). Furthermore, studies have reported that the AXL bypass activation pathway is also involved in mediating resistance of NSCLC cells to first- and second-generation ALK TKIs (126–128). However, currently, there are no studies reporting the role of AXL bypass signaling pathway activation in resistance to third-generation ALK TKIs and ROS1 TKIs.

In conclusion, AXL bypass signaling activation is one of the important reasons for resistance to NSCLC EGFR TKIs and ALK TKIs. Combining AXL inhibitors with EGFR/ALK TKIs may provide a new treatment strategy for patients with TKI resistance mediated by AXL bypass activation.

### 3.4 IGF-1R activation-mediated TKI resistance

#### 3.4.1 Overview of the IGF-1R gene

The IGF-1R gene is located on chromosome 15 (15q26) in humans and spans approximately 315 kb, containing 21 exons (129). The protein encoded by the IGF-1R gene, IGF-1R, is a transmembrane RTK belonging to the insulin receptor (INSR) family, composed of extracellular ligand-binding domains, a transmembrane domain, and intracellular domains near the membrane and tyrosine kinase domains (129, 130). When IGF-1R binds to its ligand, insulin-like growth factor (IGF), receptor structural rearrangements promote transphosphorylation, activating downstream MAPK/ERK, PI3K/AKT, and other cascading signaling pathways, thereby regulating cellular activities such as proliferation, growth, differentiation, and apoptosis (129–131). Recent studies have reported that IGF-1R also mediates resistance to NSCLC TKIs through bypass activation pathways (132–134).

#### 3.4.2 NSCLC TKI Resistance Mediated by the IGF-1R Bypass Activation Pathway

In preclinical models, abnormal activation of the IGF-1R signaling pathway is considered one of the mechanisms by which NSCLC cells develop resistance to first- and second-generation EGFR TKIs via bypass pathways. Co-administration of EGFR TKIs and IGF-1R inhibitors effectively suppresses NSCLC cells resistant through this bypass activation pathway (133–137). For instance, in gefitinib-resistant NSCLC cells A431, the loss of expression of insulin-like growth factor binding proteins (IGFBP) 3 and 4 leads to activation of the IGF-1R/PI3K/AKT pathway. When the EGFR signaling pathway is inhibited, the PI3K/AKT signaling pathway remains activated, allowing NSCLC cells to continue growing and proliferating (132). Additionally, studies have confirmed that abnormal activation of the IGF-1R signaling pathway also contributes to resistance of NSCLC cells to the third-generation EGFR TKI osimertinib (132).

In a study by Lovly et al. (46), immunohistochemical staining of five samples from crizotinib-resistant ALK fusion NSCLC patients revealed activation of IGF-1R in four cases. Preclinical models demonstrated that IGF-1R can mediate crizotinib resistance in ALK fusion NSCLC cells by sustaining activation of the PI3K/AKT signaling pathway. Inhibiting IGF-1R can restore sensitivity of NSCLC cells to crizotinib. Similar conclusions were drawn in the study by Wilson et al. (138) Furthermore, studies have reported IGF-1R-mediated resistance of ALK fusion NSCLC cells to the second-generation ALK TKI ceritinib. Co-administration of ceritinib and IGF-1R inhibitors can reverse resistance in these cells (82). These findings indicate that IGF-1R bypass signaling activation is one of the reasons for resistance of ALK fusion NSCLC to first- and second-generation ALK TKIs. Co-administration of IGF-1R inhibitors with first-generation ALK TKIs may offer further treatment possibilities for patients acquiring resistance through this bypass pathway. However, whether this bypass signaling activation mediates resistance to third-generation ALK TKIs and ROS1 TKIs has not been reported in relevant studies.

### 3.5 NSCLC TKI resistance mediated by HER2/HER3 bypass signaling pathway

The HER2 gene, also known as ERBB2, is located on chromosome 17 (17q21) in humans (139). The HER2 protein encoded by the HER2 gene is a transmembrane glycoprotein of approximately 185 kDa and is the second member of the ErbB family of RTKs (139). The HER3 gene, or ERBB3 gene, is located on the long arm of chromosome 12 (12q13) in humans (140). The HER3 protein encoded by the HER3 gene is the third member of the ErbB family of RTKs (140). Structurally, both HER2 and HER3 consist of extracellular ligand-binding domains, transmembrane domains, and intracellular tyrosine kinase domains (141, 142). HER2 is considered an “orphan receptor” as it lacks known ligands to activate downstream signaling pathways directly, though it consists of tyrosine kinase domain. Instead, HER2 forms heterodimers with other ErbB family members to activate downstream signaling pathways (142, 143). HER3 is considered a “dead kinase.” Although it can bind to multiple ligands, its intrinsic tyrosine kinase activity is impaired. It must form heterodimers with other members of the ErbB family to activate downstream signaling pathways (142). Heterodimers of HER2/HER3 are considered the most potent dimers within the ErbB family due to HER2 being the preferred dimerization partner for all ErbB family members, with HER3 being the preferred partner for HER2 (144, 145). Upon ligand binding, HER3 undergoes conformational changes allowing dimerization, thereby activating downstream PI3K/AKT, MAPK/ERK, and other cascading signaling pathways, regulating cell growth, proliferation, and resistance (142, 145, 146).

In a study, about 12% of NSCLC patients resistant to EGFR TKIs were found to have HER2 amplification, whereas the incidence of HER2 amplification in untreated NSCLC patients was only 1%, suggesting that HER2 amplification may be involved in the resistance process to EGFR TKIs. Further *in vitro* experiments confirmed that HER2 amplification activated downstream AKT and ERK signaling pathways and continued to activate them even when the EGFR signaling pathway was inhibited, leading to NSCLC cell resistance to EGFR TKIs (47). Additionally, Yu et al. (63) found that approximately 13% of NSCLC patients developed HER2 gene amplification after resistance to gefitinib or erlotinib. Besides mediating resistance to first- and second-generation EGFR TKIs, HER2 gene amplification has also been shown to mediate resistance to third-generation EGFR TKIs through bypass activation pathways (147, 148). When osimertinib was used as second-line treatment, the incidence of HER2 gene amplification in osimertinib-resistant samples was about 3% (74), while it was approximately 2% when osimertinib was used as first-line treatment (75). Studies have also reported that HER2 gene amplification can mediate primary resistance of NSCLC patients to osimertinib. Furthermore, in a study by Zhang et al. (68), it was found that approximately 3% of NSCLC patients developed HER2 gene amplification after resistance to abivertinib, another third-generation EGFR TKI. It is worth noting that the frequency of HER2 gene amplification in resistance to first-generation EGFR TKIs is significantly higher than in resistance to third-generation EGFR TKIs, which is contrary to the frequency of resistance to EGFR TKIs mediated by MET gene amplification. There is currently no relevant research to explain this phenomenon.

In addition to its role in EGFR TKI resistance, activation of the HER2/HER3 signaling pathway can also mediate resistance of NSCLC cells to ALK TKIs (149). Wilson et al. (127) found that treatment of ALK fusion NSCLC cell lines (H3122, H2228, and MGH006) with recombinant NRG1 protein (ligand of HER3) resulted in resistance to the ALK TKI TAE684, which was eliminated by co-administration of TAE684 and the HER2 inhibitor lapatinib, demonstrating that the ligand of HER3, NRG1, can mediate resistance of ALK fusion NSCLC to ALK TKIs through the activation of the HER3/HER2 signaling pathway. Another study also confirmed that the HER2 and HER3 signaling pathways were activated in TAE684-resistant ALK fusion NSCLC cells (150), mediating resistance to TAE684. Additionally, in NSCLC cells resistant to ceritinib, another second-generation ALK TKI, increased expression of HER3 and its ligand neuregulin-1 (NRG1) was observed. The upregulated HER3 protein continued to activate downstream AKT and ERK signaling pathways even when the ALK signaling pathway was inhibited, enabling cells to evade the cytotoxic effects of ceritinib (82). In clinical studies, Minari et al. (149) reported a case of ALK fusion NSCLC patient who developed HER2 gene amplification after treatment with first- and second-generation ALK TKIs. Furthermore, research has found that activation of the HER2 signaling pathway mediates resistance of NSCLC cells with ROS1 fusion to ROS1 TKIs (151).

### 3.6 NSCLC TKI resistance mediated by key gene mutations in cascade signaling pathways

The RAS/RAF/MAPK/ERK and PI3K/AKT pathways are considered the two most common cascade signaling pathways involved in bypass activation, and mutations or amplifications in key genes in these two pathways can mediate NSCLC TKI resistance (31, 32, 34, 152).

#### 3.6.1 NSCLC TKI resistance mediated by key gene mutations in the ras/raf/mapk/erk cascade signaling pathway

Mutations in genes such as KRAS, NRAS, BRAF, and MAP2K1 have been reported to mediate resistance to EGFR TKIs within the RAS/RAF/MAPK/ERK cascade signaling pathway. Eberlain et al. (153) identified the NRAS E63K mutation involved in resistance to osimertinib in NSCLC cell lines resistant to osimertinib. Both *in vitro* and *in vivo* experiments demonstrated that co-administration of osimertinib and the MEK inhibitor selumetinib could reverse the resistance mediated by the NRAS E63K mutation. Ortiz-Cuaran et al. (79) observed the KRAS G12S mutation in patients resistant to osimertinib as second-line treatment. Other KRAS mutations, such as G12D, G13D, Q61R, and Q61, have also been detected in patients resistant to osimertinib (72, 73, 154). KRAS G12D mutation or KARS gene amplification has also been reported in NSCLC patients resistant to MET inhibitors and RET inhibitors (77, 98). Furthermore, approximately 3% of patients resistant to osimertinib carry the BRAF V600E mutation (74, 75). *In vitro* experiments have confirmed that the BRAF V600E mutation mediates resistance of NSCLC cells to osimertinib, and co-administration of the BRAF inhibitor encorafenib can restore sensitivity of resistant cells to osimertinib (155). Additionally, research has found that the BRAF G469A mutation can mediate acquired resistance of NSCLC cells to osimertinib (156). Moreover, mutations such as MAP2K1 K57N, BRAF G15V, and NRAS A155T have been discovered in NSCLC resistant to second-generation ALK TKIs (157, 158). Thus, key gene mutations in the RAS/RAF/MAPK/ERK cascade signaling pathway play a significant role in NSCLC TKI resistance.

#### 3.6.2 NSCLC TKI resistance mediated by key gene mutations in the pi3k/akt cascade signaling pathway

Within the PI3K/AKT cascade signaling pathway, mutations in the PIK3CA gene are the most frequently reported genetic alterations mediating NSCLC TKI resistance. PIK3CA gene mutations have been reported in NSCLC resistant to first-, second-, and third-generation EGFR TKIs (64, 159). In clinical samples of NSCLC resistant to osimertinib as first- or second-line treatment, the incidence of PIK3CA gene mutations can reach 4%-11%, including mutations such as PIK3CA E545K, E542K, R88Q, N345K, and E418K (72–75). The osimertinib resistance mediated by the PIK3CA E545K mutation has been confirmed *in vitro* (73). Similarly, mutations in the PIK3CA gene have also been found in samples resistant to second-generation ALK TKIs (157). Thus, PIK3CA gene mutations play an important role in mediating NSCLC TKI resistance.

### 3.7 Others

Kim et al. (160) found FGFR1 gene amplification and increased expression of Fibroblast Growth Factor 2 (FGF2) in NSCLC patients resistant to osimertinib, suggesting a potential association between the FGFR1 signaling pathway and acquired resistance to osimertinib. Additionally, FGFR3 gene amplification was observed in NSCLC samples resistant to the ROS TKI crizotinib (151). These studies suggest that aberrant activation of the FGFR signaling pathway may be one of the mechanisms by which NSCLC develops acquired resistance to TKIs through bypass pathways (161, 162).

Furthermore, fusion of driver genes can also activate bypass signaling pathways to mediate acquired resistance of NSCLC to TKIs. Studies have reported that 3%-10% of patients resistant to the third-generation EGFR TKI osimertinib develop resistance through driver gene fusions. Reported fusion genes associated with resistance include MET-UBE2H, FGFR3-TACC3, RET-ERC1, CCDC6-RET, KIF5B-RET, NTRK1-TPM3, NCOA4-RET, GOPC-ROS1, AGK-BRAF, ESYT2–BRAF, PLEKHA7-ALK, and EML4-ALK, among others (70, 72–75, 163–167).

Additionally, KIT gene mutations (KIT D816G) and gene amplifications have been separately reported to mediate resistance to ROS1 TKIs and ALK TKIs (43, 151, 168).

## 4 Current treatment strategies for overcoming bypass pathway-mediated resistance

In TKI resistance mediated by bypass pathways, the bypass signaling pathway can remain activated even when the signaling pathway driven by the original driver gene is inhibited. Therefore, simultaneously inhibiting both the bypass signaling pathway and the signaling pathway driven by the original driver gene may overcome TKI resistance mediated by bypass pathways. Currently, numerous phase 1 and phase 2 clinical trials are investigating treatment strategies for NSCLC patients with TKI resistance mediated by bypass pathway activation, and some studies have already achieved promising results (see **Table 4**).

**Table 4:** Ongoing clinical trials targeting bypass activation pathways.

Drug	Clinical Trial Phase	Patient Enrollment	Start Date	Results	Clinical Trial Registration Number
Targeting MET Pathway-Mediated NSCLC TKI Resistance					
Capmatinib + Erlotinib	1/2	NSCLC patients resistant to Erlotinib	2008	Response rate: 0%	NCT00596648
Capmatinib + Erlotinib	2	NSCLC patients resistant to Gefitinib or Erlotinib with activated c-MET signaling pathway	2012	Objective response rate: 27%	NCT01610336
Capmatinib + Gefitinib	1	NSCLC patients resistant to Erlotinib with activated c-MET signaling pathway	2013	Partial response rate: 16.7%	NCT01911507
Tepotinib + Gefitinib	1/2	NSCLC patients resistant to Osimertinib with c-MET gene amplification	2013	Objective response rate: 45%	NCT01982955
Savolitinib + Osimertinib	1	EGFR TKI-resistant NSCLC patients with MET gene amplification	2014	Objective response rate: 48%	NCT02143466
Telisotuzumab vedotin + Osimertinib	1	Osimertinib-resistant NSCLC patients with c-MET positivity	2014	Ongoing	NCT02099058
Amivantamab	1	EGFR TKI-resistant NSCLC patients with MET gene amplification or mutation	2015	Ongoing	NCT02609776
Savolitinib + Gefitinib	1	First or second-generation EGFR TKI-resistant NSCLC patients with MET gene amplification	2015	Objective response rate: 25%	NCT02374645
Selpercatinib + Crizotinib	1/2	Selpercatinib-resistant NSCLC patients with MET gene amplification	2017	Response rate: 100%	NCT03157129
Telisotuzumab Vedotin (ABBV-399)	2	NSCLC patients with c-MET positivity after treatment resistance	2018	Ongoing	NCT03539536
Savolitinib + Osimertinib	2	Osimertinib-resistant NSCLC patients with c-MET positivity	2018	Ongoing	NCT03778229
Tepotinib + Osimertinib	2	First or second-generation EGFR TKI-resistant NSCLC patients with MET gene amplification	2019	Ongoing	NCT03940703
Savolitinib + Osimertinib	2	Osimertinib-resistant NSCLC patients with c-MET positivity	2019	Ongoing	NCT03944772
Amivantamab+Lazertinib	1	Osimertinib-resistant NSCLC patients	2020	Ongoing	NCT04077463
Volitinib+Osimertinib	2	Osimertinib-resistant NSCLC patients with MET gene amplification	2020	Ongoing	NCT04606771
Glumetinib+Osimertinib	1/2	EGFR TKI-resistant NSCLC patients with MET gene amplification	2020	Ongoing	NCT04338243
Targeting HER2/HER3 Pathway Mediated NSCLC TKI Resistance					
Pertuzumab+Trastuzumab	2	TKI-resistant patients mediated by HER2 gene amplification	2017	Ongoing	NCT03297606
Trastuzumab emtansine+Osimertinib	2	EGFR TKI-resistant NSCLC patients with activated HER2 signaling pathway	2018	Ongoing	NCT03784599
Patritumab Deruxtecan	1	NSCLC patients resistant to EGFR TKI	2017	Ongoing	NCT03260491
Patritumab Deruxtecan	2	NSCLC patients resistant to EGFR TKI	2020	Ongoing	NCT04619004
Patritumab Deruxtecan+Osimertinib	1	Osimertinib-resistant NSCLC patients	2020	Ongoing	NCT04676477
Pertuzumab+Necitumumab+Osimertinib	1/2	Osimertinib-resistant NSCLC patients	2020	Ongoing	NCT04285671
Targeting AXL Pathway Mediated NSCLC TKI Resistance					
Bemcentinib+Erlotinib	1/2	Erlotinib-resistant NSCLC patients	2015	Ongoing	NCT02424617
Dubermatinib	1	NSCLC patients resistant to EGFR TKI	2016	Ongoing	NCT02729298
DS-1205c+Osimertinib	1	NSCLC patients resistant to EGFR TKI	2017	Ongoing	NCT03255083
DS-1205c+Gefitinib	1	NSCLC patients resistant to EGFR TKI	2018	Ongoing	NCT03599518
Targeting RAS/RAF/MEK/ERK and PI3K/AKT Pathways Mediated NSCLC TKI Resistance					
BKM120+Gefitinib	1	EGFR TKI-resistant NSCLC patients with activated PI3K signaling pathway	2012	Median progression-free survival: 2.8 months	NCT01570296
Simeprevir+Osimertinib	1	EGFR TKI-resistant NSCLC patients	2014	Ongoing	NCT02143466
Cobimetinib+Ceritinib	1/2	ALK TKI-resistant NSCLC patients	2017	Ongoing	NCT03087448
Cobimetinib+Alectinib	1/2	Alectinib-resistant NSCLC patients	2017	Ongoing	NCT03202940
Cobimetinib+EGF816	1	EGFR TKI-resistant NSCLC patients	2018	Ongoing	NCT03516214
Sotorasib	2	TKI-resistant NSCLC patients	2020	Ongoing	NCT04625647
Targeting Other Pathways Mediating NSCLC TKI Resistance					
Xentuzumab+Afatinib	1	EGFR TKI-resistant NSCLC patients	2014	Partial response rate: 13%	NCT02191891
Amivantamab	1/2	EGFR TKI-resistant NSCLC patients	2018	Ongoing	NCT03706287
Alectinib+Osimertinib	2	Osimertinib-resistant NSCLC patients with ALK fusion	2019	Ongoing	NCT03944772
Selpercatinib+Osimertinib	2	Osimertinib-resistant NSCLC patients with RET fusion	2019	Ongoing	NCT03944772
Amivantamab	2	EGFR TKI-resistant NSCLC patients	2020	Ongoing	NCT04619563
Sunvozertinib	2	EGFR TKI-resistant NSCLC patients	2022	Ongoing	NCT03974022

### 4.1 Targeting MET bypass activation in NSCLC TKI resistance

Currently, over 50 MET-targeted drugs are under research or have entered clinical use, mainly including anti-HGF antibodies, anti-c-MET antibodies, and MET inhibitors, among which MET inhibitors are the most extensively studied and applied c-MET targeted drugs. Presently, there are four FDA-approved small molecule inhibitors targeting c-MET, namely crizotinib (169), cabozantinib (170), capmatinib (171), and tepotinib (172). Among these, crizotinib and cabozantinib are multitargeted drugs (including ALK and ROS1), initially not marketed with c-Met as a therapeutic target, while capmatinib and tepotinib were approved with c-MET as the therapeutic target and are highly selective c-Met inhibitors. Brigatinib also belongs to the new generation of ALK inhibitors and has been approved for use in patients with ALK-positive metastatic NSCLC who have undergone disease progression on crizotinib therapy or who cannot tolerate crizotinib (173). For ROS1 translocation malignancies that have developed resistance to crizotinib, cabozantinib may be effective. Ceritinib has also been shown to be efficacious, but may not be able to overcome the problem of acquired resistance to crizotinib (174). The MET inhibitors glesatinib, savolitinib and the EGFR/MET bispecific antibody amivantamab are also in clinical trials. Worth mentioning is the domestically produced highly selective c-MET inhibitor volitinib (Savolitinib), which has entered the FDA New Drug Application (NDA) stage, representing a promising c-MET inhibitor.

Some preclinical studies have demonstrated that in NSCLC resistant cells mediated by MET gene amplification, the combination of MET inhibitors and EGFR TKIs can reverse the resistance of resistant cells. Simultaneously, in clinical reports, the combination of crizotinib and osimertinib has been proven to be an effective treatment strategy for osimertinib-resistant patients with MET gene amplification. In clinical research, in a phase 1b/2 trial, 47% of EGFR TKI-resistant NSCLC patients with MET gene amplification and 32% of EGFR TKI-resistant patients with MET overexpression showed objective responses to the combination treatment of the MET inhibitor capmatinib and gefitinib (175). In another phase 1b trial of the combination of the MET inhibitor volitinib and gefitinib, 52% of EGFR TKI-resistant NSCLC patients with MET gene amplification exhibited objective responses to the combination therapy (176). In the subsequent INSIGHT study, 67% of EGFR TKI-resistant NSCLC patients with MET gene amplification showed objective therapeutic responses to the combination of the MET inhibitor tepotinib and gefitinib (177). In the phase 1b trial of the TATTON study, 64% of NSCLC patients resistant to first- or second-generation EGFR TKIs and with MET gene amplification demonstrated objective responses to the combination of volitinib and osimertinib, while only 30% of patients resistant to third-generation EGFR TKIs and with MET gene amplification showed objective responses to this combination therapy (178). Based on the results of the TATTON study, the SAVANNAH study and the ORCHARD study are exploring the use of volitinib in combination with osimertinib for the treatment of osimertinib-resistant NSCLC patients with MET gene amplification (NCT03778229 and NCT03944772). The savolitinib + osimertinib combination represents a promising therapy in patients with MET-amplified/overexpressed, EGFRm advanced NSCLC with disease progression on a prior EGFR-TKI (NCT02143466) (179). The combination of osimertinib+chemotherapy in EGFR-Mutated Advanced NSCLC not only helps patients with survival rates and disease progression but also has a higher response rate and longer response duration (180). Additionally, the INSIGHT 2 study is investigating the combination of the MET inhibitor tepotinib with osimertinib for the treatment of EGFR TKI-resistant NSCLC patients with MET amplification (NCT03940703).

Currently, sporadic case reports exist regarding the combined use of MET inhibitors with ALK TKIs or RET TKIs, but there are no clinical studies specifically targeting MET inhibitors in combination with ALK TKIs or RET TKIs. Gouji et al. (181) reported a case of an ALK fusion NSCLC patient who developed MET gene amplification after resistance to the second-generation ALK TKI alectinib, achieving a response to treatment with crizotinib; however, the patient died after 7 months of crizotinib treatment. Dagogo-Jack et al (76). reported a case of an ALK fusion NSCLC patient who showed significant MET gene amplification after resistance to alectinib, achieving a response after treatment with crizotinib; however, the tumor recurred after 10 weeks of treatment. Dagogo-Jack et al. (76) also reported another case of an ALK fusion NSCLC patient who developed MET gene amplification after resistance to lorlatinib, achieving tumor control after treatment with lorlatinib in combination with crizotinib, but the tumor progressed again after 3 months of treatment. In the study by Rosen et al. (182), four NSCLC patients treated with the RET inhibitor selpercatinib were found to have MET gene amplification after resistance of selpercatinib treatment, all showing varying degrees of therapeutic response to crizotinib combined with selpercatinib; however, tumors recurred in all patients after 3.5 to 10 months. Larger clinical trials are needed to confirm the efficacy of ALK or RET TKIs in combination with MET inhibitors in NSCLC patients with MET gene amplification who are resistant to ALK or RET TKIs.

### 4.2 Targeting HER2/HER3 bypass activation in NSCLC TKI resistance

Several small molecule inhibitors targeting HER2 or therapeutic antibodies have been FDA-approved, while there are currently no marketed drugs targeting HER3. FDA-approved small molecule inhibitors targeting HER2 include lapatinib (183), afatinib (184), neratinib (185), and dacomitinib (17); therapeutic antibodies targeting HER2 include Herceptin, trastuzumab (186), Phesgo, pertuzumab (187), and Enhertu (trastuzumab deruxtecan) (188).

Currently, multiple trials are investigating the efficacy of HER2/HER3 inhibitors or antibodies in patients with NSCLC TKI resistance driven by HER2/HER3 signaling pathway activation, but standard treatment regimens have not been established. In NSCLC patients with HER2 gene amplification, trastuzumab-DM1 (trastuzumab emtansine) has shown promising efficacy in phase 2 clinical trials (189), and the ongoing TRAEMOS trial is investigating the therapeutic effect of T-DM1 combined with osimertinib in osimertinib-resistant patients with HER2 amplification (NCT03784599). In a study on patients with advanced NSCLC harboring HER-2 mutations, the efficacy and pharmacotoxicity of pyrotinib were analyzed, thereby expanding treatment options for rare gene mutations (NCT03574402) (190). The CAPTUR trial is also investigating the combination of trastuzumab and pertuzumab to overcome resistance mediated by HER2 gene amplification (NCT03297606). Additionally, a phase 1 trial is investigating the therapeutic effect of the HER3-targeting antibody-drug conjugate U3-1402 (patritumab deruxtecan) combined with osimertinib in osimertinib-resistant NSCLC patients (NCT04676477).

### 4.3 Targeting AXL bypass activation in NSCLC TKI resistance

Preclinical studies have confirmed that combination therapy targeting AXL can effectively reverse NSCLC TKI resistance mediated by AXL bypass activation. In ongoing clinical trials, three AXL inhibitors (bemcentinib, dubermatinib, and DS-1205c) are being used alone or in combination with EGFR TKIs to treat EGFR TKI-resistant NSCLC patients (NCT02424617, NCT02729298, NCT03255083, NCT03599518). However, unfortunately, these AXL inhibitors have not yet been FDA-approved for clinical use, and there is currently no standard treatment regimen for NSCLC AKI resistance mediated by AXL bypass activation.

### 4.4 Targeting cascade signaling pathways in NSCLC TKI resistance

Multiple drugs targeting cascade signaling pathways involved in NSCLC TKI resistance are currently being investigated in clinical trials. In studies targeting the PI3K/AKT cascade signaling pathway mediated NSCLC TKI resistance treatment, the PI3K inhibitor BKM120 combined with gefitinib was used to treat EGFR TKI-resistant NSCLC patients with PI3K signaling pathway activation, but unfortunately, the median progression-free survival was only 2.8 months (191). The novel PI3K inhibitor alpelisib was FDA-approved in 2019 for the treatment of PIK3CA-mutated breast cancer, and considering the high incidence of PIK3CA mutations in NSCLC TKI resistance (2%-11%), future clinical trials may use next-generation PI3K inhibitors to explore their efficacy in NSCLC TKI-resistant patients with PIK3CA mutations. Adagrasib is a related molecule that the FDA has also approved for patients with locally advanced or metastatic NSCLC with the KRAS G12C mutation who have received at least one prior systemic therapy (192).

In the treatment of NSCLC TKI resistance mediated by the RAF/RAS/MEK/ERK cascade signaling pathway, the MEK inhibitors selumetinib (190, 192), trametinib (193), and cobimetinib (194) have been FDA-approved. The KRAS inhibitor sotorasib (AMG 510) (195) has also received FDA breakthrough therapy designation and real-time oncology review qualification for the treatment of NSCLC patients with KRAS G12C mutations. A phase I study enrolling 137 patients with advanced KRAS-mutant cancers showed that treatment with the covalent KRAS G12C inhibitor divarasib (GDC-6036) had an ORR of 53% and a median PFS of 13.1 months in 60 patients with NSCLC (196). There are few promising treatments for non-G12C KRAS-mutant NSCLC. A phase III trial combining the oral MEK inhibitor selumetinib with docetaxel did not find a beneficial MEK inhibitor treatment strategy (197). Ongoing clinical trials include trametinib in combination with ceritinib for the treatment of ALK TKI-resistant NSCLC patients (NCT03087448), cobimetinib in combination with alectinib for the treatment of alectinib-resistant NSCLC patients (NCT03202940), selumetinib in combination with osimertinib for the treatment of EGFR TKI-resistant NSCLC patients (NCT02143466), and sotorasib for the treatment of TKI-resistant NSCLC patients (NCT04625647).

### 4.5 Other

RET gene fusion is one mechanism of NSCLC TKI bypass activation resistance, and currently, the FDA has approved two RET inhibitors, pralsetinib (BLU-667) (198) and selpercatinib (LOXO-292) (199), for the treatment of RET fusion-positive NSCLC patients. In clinical reports, two NSCLC patients resistant to osimertinib due to RET gene fusion achieved clinical remission after receiving pralsetinib in combination with osimertinib (70). Additionally, a clinical trial is investigating selpercatinib in combination with osimertinib for the treatment of osimertinib-resistant patients with RET gene fusion (NCT03944772).

Combination chemotherapy presented good results in patients with advanced NSCLC in which EGFR mutations were found. A non-blind randomized trial enrolling 557 patients with advanced NSCLC carrying EGFR mutations found that the addition of platinum and pemetrexed chemotherapy to ositinib improved PFS (180). For primary patients with locally advanced or metastatic NSCLC harboring an EGFR mutation (exon 19 deletion or L858R mutation), treatment with amivantamab + lazertinib resulted in a better PFS improvement than ositinib alone (200).

Furthermore, clinical trials are underway for the combination of alectinib with osimertinib for the treatment of osimertinib-resistant NSCLC patients with ALK fusion (NCT03944772), IGF-1R therapeutic antibody in combination with afatinib for the treatment of EGFR TKI-resistant NSCLC patients (NCT02191891), and FGFR inhibitors for the treatment of EGFR TKI-resistant NSCLC patients (NCT03706287, NCT04619563). Oral inhibitors have been shown to be particularly effective and less toxic for the initial treatment of patients with advanced NSCLC who have concomitant driver mutations and who are older or in poorer health. This approach is particularly useful in patients with EGFR mutations, BRAF mutations, ALK fusion oncogenes, or ROS1 translocations, subject to ongoing discovery of other targetable mutations.

## 5 Conclusion and outlook

### 5.1 Conclusion

The use of TKI drugs has significantly improved the prognosis of NSCLC patients carrying driver genes. However, the emergence of resistance poses new challenges to further extending patient survival, and a deeper understanding and study of the molecular mechanisms of TKI resistance will be the foundation for overcoming TKI resistance. In NSCLC, the molecular mechanisms of TKI resistance include secondary mutations of driver genes, bypass signal activation, and histological transformation. Secondary mutations of driver genes can be overcome by developing new generations of TKI drugs, while resistance mechanisms caused by bypass signal activation are more complex. In TKI resistance caused by bypass signal activation, although TKI drugs inhibit downstream signaling pathways of driver genes, RTKs can continuously activate key signaling pathways within tumor cells through alternative pathways, thereby sustaining tumor cell growth and proliferation, leading to resistance to TKI drugs. Inhibiting activated bypass signals and the original driver gene signaling pathway is the basic concept for treating bypass activation-mediated TKI resistance. Based on this concept of combination therapy, several clinical trials are currently underway to treat patients resistant to bypass signal activation, although all trials are in early stages, some have yielded promising preliminary results, which will provide more treatment options for NSCLC TKI-resistant patients.

### 5.2 Outlook

In recent years, based on next-generation sequencing of circulating tumor DNA (ctDNA) detection, dynamic monitoring of molecular genetic changes occurring during TKI treatment of tumor patients through tumor rebiopsy can timely discover changes in bypass signals leading to resistance, such as MET gene amplification, HER2 gene amplification, KRAS gene mutation, PIK3CA gene mutation, etc. (69, 180, 201–203). The FDA has approved a number of ctDNA tests for identifying patients with positive EGFR mutations (204, 205), and one of these tests uses NGS to identify abnormalities in 55 additional genes (205). Although tissue availability is limited, somatic gene alteration testing prior to treatment of advanced NSCLC remains recommended. In a trial of adult patients with advanced NSCLC that used blood liquid biopsies to assess circulating tumor DNA to determine whether patients with ROS1-positive NSCLC were treated with the entrectinib, the assay resulted in an 81% objective response rate to entrectinib, which is consistent with the results of a previous study using a tissue-based assay to identify ROS1 fusions (206). The use of liquid biopsies to assess other molecular abnormalities may become more common as more data become available.

Through this approach, on one hand, more precise individualized tumor treatment plans can be formulated for patients, and on the other hand, effective treatment can be given to patients in a timely manner to prevent further occurrence and development of resistance. The ongoing ELIOS trial is such a study, in which NSCLC patients receiving osimertinib as first-line treatment will obtain tumor biopsy samples and plasma samples before treatment, during treatment, and after treatment resistance, and compare and analyze the genetic variations of tissue samples and plasma samples through next-generation sequencing technology to reveal the role of liquid biopsy technology in resistance monitoring (NCT03239340). Meanwhile, the complementary ORCHARD clinical trial aims to study the treatment plan for NSCLC patients receiving osimertinib as first-line treatment after acquired resistance. In this innovative trial platform, resistant patients will be allocated to corresponding treatment cohorts based on their different tumor molecular characteristics and different resistance mechanisms, including cohorts of wolinib combined with osimertinib, gefitinib combined with osimertinib, alectinib combined with osimertinib, and selpercatinib combined with osimertinib, etc. (NCT03944772). Integrating the results of these two trials will provide more precise, effective, and personalized treatment for NSCLC patients.

In addition, the concept of “Drug-Tolerant Persister (DTP) cells” has been proposed, refreshing researchers’ understanding of the mechanisms of tumor resistance (207). A small proportion of tumor cells (0.3%-5%) can survive by adopting a reversible drug-tolerant state when facing TKI treatment, and under the continuous action of drugs, eventually acquire permanent resistance mechanisms based on genetic changes (207). Turke et al.’s (61) study found that a small number of cells (<1%) with MET gene amplification could be detected in samples from NSCLC patients before using EGFR TKIs, while a large number of cells with MET amplification were present after EGFR TKI resistance, indicating that the small fraction of cells with MET gene amplification before treatment ultimately led to resistance. In Taniguchi et al.’s (208) study, DTP cells obtained after treating PC-9 cells with osimertinib for 9 days showed significantly increased expression of AXL, and resistance was significantly higher than that of sensitive cells, while combined use of osimertinib and AXL inhibitors could prevent the generation of DTP cells. Wang et al.’s (209) study also found that although NSCLC cells with low expression of AXL were more sensitive to osimertinib, there still existed a small group of cells activated by IGF-1R signaling that were resistant to osimertinib treatment, while the use of IGF-1R inhibitors could prevent the generation of DTP cells. These studies suggest that early targeting of DTP cells may delay the occurrence of NSCLC TKI resistance, thereby increasing patient survival. In an ongoing clinical trial, researchers are adding c-MET inhibitors (APL-101) for combination therapy in NSCLC patients receiving first-line osimertinib treatment between 8 and 12 weeks before the occurrence of resistance, to observe whether this treatment regimen can delay the onset of resistance in patients (NCT04743505).

In summary, by utilizing liquid biopsy techniques to identify resistance mechanisms early, implementing individualized targeted therapies against these mechanisms at an early stage, and preemptively targeting drug-tolerant persister (DTP) cells, NSCLC patients may achieve more durable and effective treatments, leading to further extensions in survival time.
